# Agroinfiltration Reduces ABA Levels and Suppresses *Pseudomonas syringae-*Elicited Salicylic Acid Production in *Nicotiana tabacum*

**DOI:** 10.1371/journal.pone.0008977

**Published:** 2010-01-29

**Authors:** Arantza Rico, Mark H. Bennett, Silvia Forcat, Wei E. Huang, Gail M. Preston

**Affiliations:** 1 Department of Plant Sciences, University of Oxford, Oxford, United Kingdom; 2 Biology Division, Imperial College London, London, United Kingdom; 3 Kroto Research Institute, The University of Sheffield, Sheffield, United Kingdom; Cairo University, Egypt

## Abstract

**Background:**

*Agrobacterium tumefaciens* strain GV3101 (pMP90) is widely used in transient gene expression assays, including assays to study pathogen effectors and plant disease resistance mechanisms. However, inoculation of *A. tumefaciens* GV3101 into *Nicotiana tabacum* (tobacco) leaves prior to infiltration with pathogenic and non-host strains of *Pseudomonas syringae* results in suppression of macroscopic symptoms when compared with leaves pre-treated with a buffer control.

**Methodology/Findings:**

To gain further insight into the mechanistic basis of symptom suppression by *A. tumefaciens* we examined the effect of pre-treatment with *A. tumefaciens* on the growth of *P. syringae*, the production of the plant signalling molecules salicylic acid (SA) and abscisic acid (ABA), and the presence of callose deposits. Pre-treatment with *A. tumefaciens* reduced ABA levels, *P. syringae* multiplication and *P. syringae*-elicited SA and ABA production, but promoted increased callose deposition. However, pre-treatment with *A. tumefaciens* did not suppress necrosis or SA production in leaves inoculated with the elicitor HrpZ.

**Conclusions/Significance:**

Collectively, these results show that inoculation of *N. tabacum* leaves with *A. tumefaciens* alters plant hormone levels and plant defence responses to *P. syringae*, and demonstrate that researchers should consider the impact of *A. tumefaciens* on plant signal transduction when using *A. tumefaciens*-mediated transient expression assays to investigate ABA-regulated processes or pathogenicity and plant defence mechanisms.

## Introduction

*Agrobacterium tumefaciens* is a soil-borne pathogen that causes crown gall disease in dicotyledoneous plants. The bacterium transfers the T-DNA region from its tumor-inducing (Ti) plasmid into plant cells, where it integrates into the plant genome. Once integrated in the plant chromosome, expression of T-DNA encoded genes elicits a hyperplastic response that results in gall formation [1], [2]. The ability of *A. tumefaciens* to transfer DNA across kingdoms has been exploited for biotechnological applications and *A. tumefaciens*-mediated transformation is routinely used to engineer plants and to express, disrupt or silence genes in functional genomic studies [3], [4]. *A. tumefaciens* can also be used in transient expression assays, where high densities of *A. tumefaciens* are infiltrated into plant tissues and transgene properties can be assayed within a few days of infiltration. Transient expression is of particular relevance in studies of gene function, protein localization and protein production [5]–[7].

*Agrobacterium-*mediated transient expression (agroinfiltration) assays are commonly carried out using *Nicotiana tabacum* (tobacco), because its leaves are easy to infiltrate and manipulate, and because *A. tumefaciens* does not have any visible effect on plant health, in contrast to the necrosis elicited by *A. tumefaciens* when it is introduced into plants such as tomato and *Arabidopsis thaliana* [8], [9]. Although *N. tabacum* does not exhibit macroscopic responses to *A. tumefaciens* during transient expression assays, numerous studies have shown that asymptomatic challenge of plants with microorganisms or with microbe-derived molecules can prime or modify plant defences [10]–[12]. It is therefore logical to hypothesise that infiltration of high densities of *A. tumefaciens* into bacterial leaves could have a significant impact on the behaviour of the plant immune system. In this work we have investigated how inoculation of *A. tumefaciens* into *N. tabacum* leaves at concentrations typically used for transient expression assays affects the production of plant hormones and plant defence responses to bacterial pathogens.

Early studies of *A. tumefaciens*-plant interactions suggested that *A. tumefaciens* did not produce elicitors that elicit plant immune responses, as flagellin and flagellin-derived peptides from *A. tumefaciens* failed to act as pathogen-associated molecular patterns (PAMPs, also known as microbial associated molecular patterns (MAMPs)), in *Arabidopsis thaliana* [13], [14]. Flagellins from other plant pathogens, such as *Pseudomonas syringae*, are recognised by *Arabidopsis* cells, resulting in ROS production and callose deposition [15], [16]. However, recent studies have provided increasing evidence that *A. tumefaciens* does elicit PAMP-triggered immunity (PTI), although the nature of the elicitors it produces is not fully understood [3], [9], [17], [18]. For example, although *Arabidopsis* does not recognise *A. tumefaciens* flagellin, *A. tumefaciens* can elicit PTI in *Arabidopsis* via recognition of elongation factor Tu (EF-Tu) by the EF-Tu receptor kinase (EFR) [9]; and via recognition of peptidoglycan (PGN) [19]. In addition, Ditt *et al*. [3] have proposed that a diffusible elicitor from *A. tumefaciens* activates basal defence responses in *Ageratum conyzoides*.

There is species-specific variation in PAMP recognition by plants, and neither flagellin nor EF-Tu account for *N. tabacum* responses to *A. tumefaciens* [9], [13]. However, *N. tabacum* has been shown to respond to the conserved RNA-binding motif RNP-1 of bacterial cold shock protein (CSP) [19], [20], which is present in *A. tumefaciens*.

The mounting evidence that *A. tumefaciens* can trigger PTI in many, and possibly all of its plant hosts supports the hypothesis that one function of T-DNA encoded proteins and other *A. tumefaciens* virulence factors is to modify and suppress plant defence responses [18]. For example, *A. tumefaciens* may use auxins not only to promote host cell division and tumor proliferation but also to suppress host defences by an antagonistic interaction with salicylate (SA)-mediated defence signalling [21]. Pruss and collaborators [17] have demonstrated that infiltration of *N. tabacum* with the oncogenic *A. tumefaciens* strain C58 elicits accumulation of miR393, a microRNA that represses auxin signalling and promotes antibacterial resistance. However, miR393 expression was repressed in *A. tumefaciens* induced tumors, which led them to speculate that miR393 is down-regulated in later stages of *A. tumefaciens* infection due to increased auxin synthesis resulting from transfer and expression of T-DNA genes.

We have observed that infiltration of *A. tumefaciens* into *N. tabacum* leaves reduces their susceptibility to bacterial pathogens such as *P. syringae* pv. tabaci (Pta) and attenuates or eliminates the hypersensitive response (HR) to the avirulent pathogen *P. syringae* pv. tomato (Pto). A similar phenomenon was reported by Pruss and collaborators [17], who observed that infiltration with a disarmed *A. tumefaciens* strain protected tobacco leaves from infection with tobacco mosaic virus (TMV). The observation that pre-treatment with a non-pathogenic bacterium elicits PTI and suppresses disease or the HR arising from subsequent inoculation of a pathogenic bacterium is not new, as previous studies have described this phenomenon with non-pathogenic strains of *Pseudomonas* [22]–[24]. However, this is a relatively uncharacterised phenomenon in the context of *A. tumefaciens*-plant interactions, despite the popularity of *A. tumefaciens* as a delivery mechanism for transient expression.

In this report we demonstrate that infiltration of *A. tumefaciens* GV3101 into *N. tabacum* leaves not only reduces necrosis associated with disease or the HR, but also suppresses *P. syringae* growth. We examine the effect of *A. tumefaciens* on plant defence responses and plant hormones and show that infiltration of *A. tumefaciens* into *N. tabacum* leaves elicits a low level of callose deposition and results in reduced abscisic acid (ABA) levels. We also show that when *A. tumefaciens-*treated leaves are challenged with *P. syringae* they display reduced salicylic acid (SA) production and increased callose deposition. This clearly demonstrates that agroinfiltration can have effects on plant signal transduction and plant defence responses that are unrelated to transient expression of specific genes. A preliminary report outlining some of these findings was presented at the 7^th^ International Conference on *Pseudomonas syringae* pathovars and related pathogens [25].

## Results

### Infiltration of *A. tumefaciens* into *N. tabacum* Leaves Prior to Inoculation with *P. syringae* Results in Reduced Macroscopic Symptoms and Reduced *P. syringae* Population Levels

**Figure 1:**
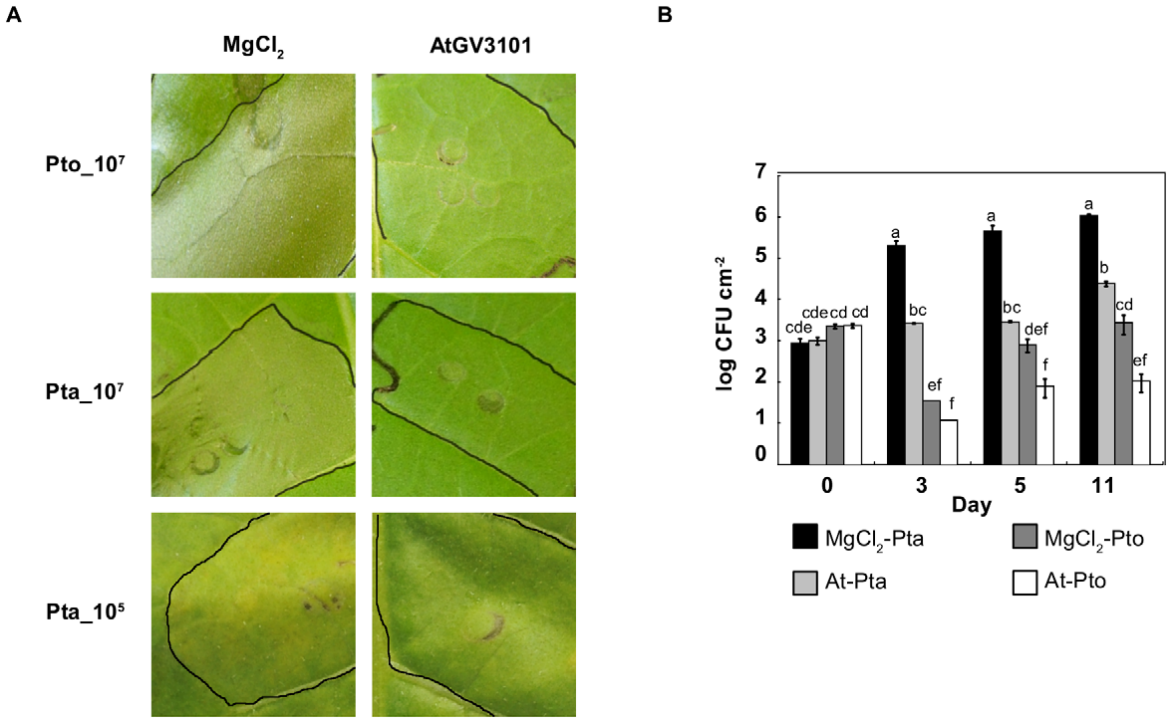
Agroinfiltration of *N. tabacum* leaves prior to *P. syringae* infection reduces macroscopic symptoms and *P. syringae* growth. **A.** *N. tabacum* leaves were infiltrated with 10 mM MgCl_2_(AS) (MgCl_2_) or *A. tumefaciens* GV3101 (AtGV3101) at 10^7^ cfu/ml. *P. syringae* pv. tabaci 11528 (Pta) and *P. s.* pv. tomato DC3000 (Pto) were infiltrated 48 hours later at 10^7^ cfu/ml. Images show infiltrated areas from representative leaves photographed 24 hours after inoculation with Pta and Pto at 10^7^ cfu/ml, (top panels) or 6 days after inoculation with Pta at 10^5^ cfu/ml (bottom panels). **B.** *N. tabacum* leaves were inoculated with 10 mM MgCl_2_(AS) or AtGV3101 as described in **A**, followed 48 hours later by inoculation with *P. syringae* at 10^5^ cfu/ml. Population densities of *P. syringae* were estimated at 0, 3, 5 and 11 days after inoculation by dilution plating of homogenised leaf extracts onto selective media. CFU, colony forming units; MgCl_2_-Pta, Pta population density after MgCl_2_(AS) treatment; MgCl_2_-Pto, Pto population density after MgCl_2_(AS) treatment; At-Pta, Pta population density after AtGV3101 treatment; At-Pto, Pto population density after AtGV3101 treatment. Data shown is the average of three replicates. Error bars show standard deviation. General Linear Model (GLM) analysis revealed statistical differences between treatments (F = 56.4244; p<0.0001; df = 15). Means with the same letter were not significantly different at the 5% confidence level based on Tukey's Honestly Significant Mean Differences (HSD) Test. The experiment was performed twice with similar results.

While performing transient expression assays using agroinfiltration to monitor protein expression and localisation in *N. tabacum* leaves infected with the pathogen *P. syringae* pv. tabaci (Pta) we observed reduced or complete elimination of necrosis in leaves infected with Pta, compared to leaves treated with the buffer control (10 mM MgCl_2_ supplemented with 0.5 mM acetosyringone (MgCl_2_(AS)), data not shown). This suggested that the *A. tumefaciens* strain used, *A. tumefaciens* GV3101 (AtGV3101), was affecting either plant signal transduction or pathogen behaviour. To systematically investigate whether disease or HR-associated necrosis induced by *P. syringae* was attenuated by the presence of AtGV3101, we inoculated AtGV3101 into *N. tabacum* leaves at 10^7^ cfu/ml and 48 hours (h) later challenged the leaves with Pta or the non-host pathogen *P. syringae* pv. tomato DC3000 (Pto) at 10^7^ cfu/ml. After 24 hours, we observed confluent necrosis in leaf panels pre-treated with MgCl_2_(AS) and then inoculated with Pto or Pta. However, pre-treatment with AtGV3101 resulted in a significant reduction in the amount of necrosis observed (Fig. 1A), indicating that AtGV3101 inhibits both the HR caused by Pto, and the necrosis associated with inoculation of high densities of Pta.

The effect of AtGV3101 on *P. syringae* growth was measured in experiments in which Pta and Pto were infiltrated into *N. tabacum* leaves at 10^5^ cfu/ml, 48 h after infiltration of AtGV3101. As expected, the population density of the non-host pathogen Pto did not increase significantly over time in either MgCl_2_(AS) or AtGV3101-pre-treated leaves (Fig. 1B). However, we observed at least a 10-fold reduction in Pto population density in AtGV3101*-*pre-treated leaves relative to MgCl_2_(AS)-pre-treated leaves at 5 and 11 days post-inoculation (Fig. 1B). In contrast, the population density of Pta did increase over time, however, AtGV3101 pre-treatment resulted in a 100-fold and 40-fold reduction in population density at 5 and 11 days post-inoculation respectively (Fig. 1B). Consistent with this, the symptoms produced by Pta in AtGV3101*-*pre-treated leaves were severely reduced or absent (Fig. 1A, bottom panel). The population density of AtGV3101 remained relatively constant throughout the experiment (data not shown). Collectively, these results show that infiltration of AtGV3101 at densities typically used for transient expression, prior to inoculation of *P. syringae*, inhibits Pta growth, and reduces the viability of Pto, even though the HR elicited by Pto is attenuated.

### *A. tumefaciens* Suppresses *P. syringae*-Elicited SA Production and SA-Mediated Defence Responses

**Figure 2:**
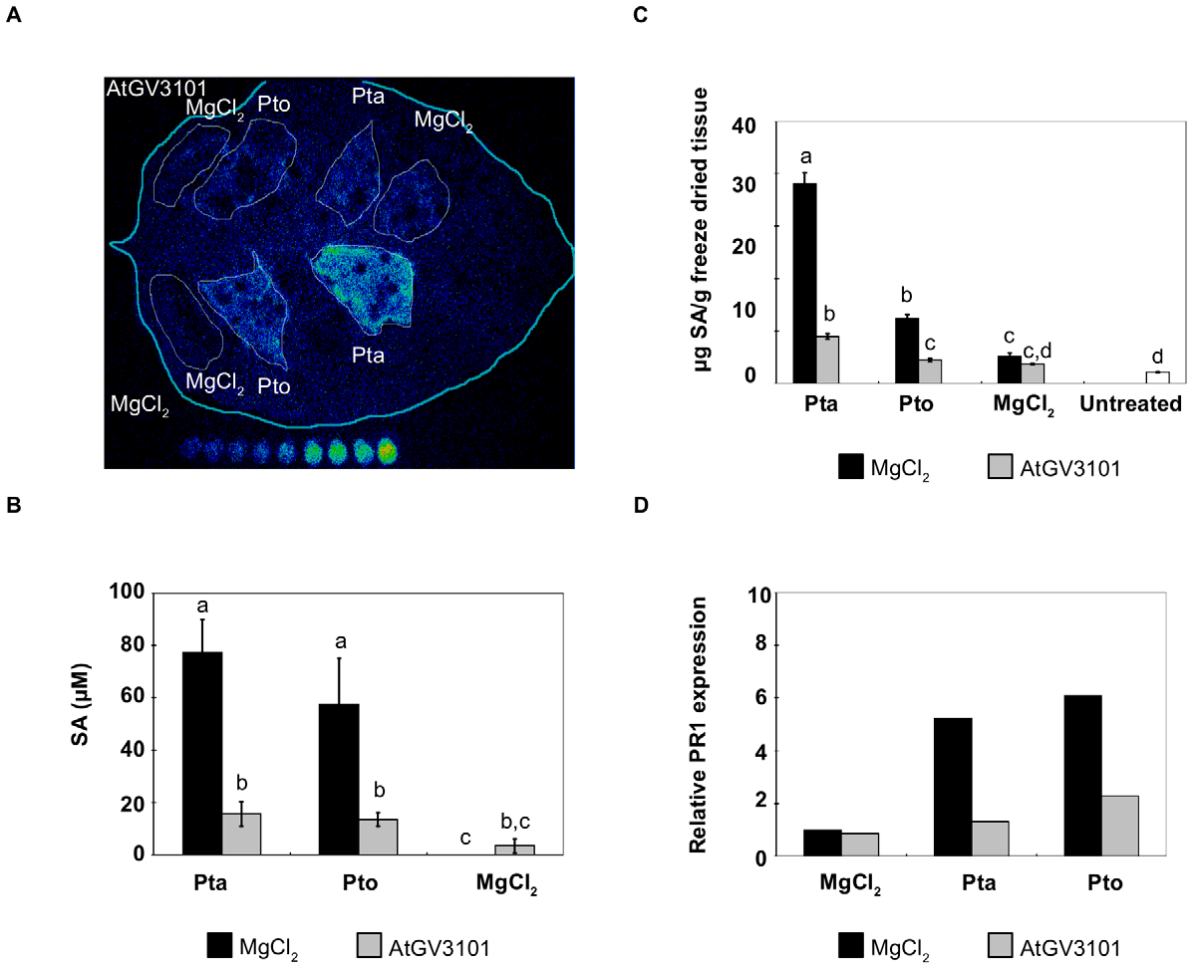
Agroinfiltration of *N. tabacum* leaves prior to *P. syringae* infection reduces *P. syringae*-elicited salicylic acid (SA) production and *PR1a* expression. In all experiments, leaves were inoculated with *A. tumefaciens* GV3101 (AtGV3101) at 10^7^ cfu/ml or MgCl_2_(AS), followed by inoculation with *P. syringae* pv. tabaci 11528 (Pta) or *P. s.* pv. tomato DC3000 (Pto) at 10^5^ cfu/ml or with MgCl_2_ after 48 hours. **A.** The leaf shown was inoculated with AtGV3101 (top panels) and MgCl_2_ (bottom panels). The SA biosensor ADPWH-*lux* was inoculated into leaves 24 hours after infiltration with *P. syringae*. SA-induced luminescence was measured one hour after biosensor inoculation using a photon-counting camera. *In vitro* SA concentration ladders were included with each leaf imaged to allow comparison of bioluminescence between separate images. **B.** Numerical SA values for leaves inoculated with ADPWH-*lux* were calculated using a calibration curve as described in Huang et al. [26]. The chart shows average SA values from three independent experiments. GLM analysis revealed statistical differences between treatments (F = 23.6361; p<0.0001; df = 5). Means with the same letter were not significantly different at the 5% confidence level based on Tukey's HSD Test. **C.** SA levels in whole leaf extracts. SA content was determined by LC/MS/MS as described by Forcat et al. [61] The graph shows average values from five independent experiments. One way ANOVA revealed statistical differences between treatments (F = 24.9968; p<0.0001; df = 6). Means with the same letter were not significantly different at the 5% confidence level based on Student's t-test. Bars indicate the standard error of the mean. **D.** *PR1a* expression relative to the housekeeping gene *EF1-α* measured by quantitative real-time PCR. The figure shows a representative experiment. Similar ratios of *PR1a* expression in agroinfiltrated leaves with respect to mock-treated leaves were observed in three separate experiments.

The reduced necrosis and pathogen growth observed in AtGV3101-treated leaves suggested that other aspects of *P. syringae-*plant interactions, such as plant defence responses, might also be affected by pre-treatment with AtGV3101. One defence response known to be up-regulated during the interaction of Pto and Pta with *N. tabacum* is the production of the defence signal SA [26]. We therefore used the SA biosensor *Acinetobacter baylyi* ADPWH-*lux* (ADPWH-*lux*) [26] to examine whether AtGV3101 suppressed *P. syringae*-elicited production of apoplastic SA. When plants were inoculated with MgCl_2_(AS) followed by *P. syringae*, both the host pathogen Pta and the non-host pathogen Pto elicited a significant increase in apoplastic SA 24 h after inoculation (77.4±12.4 and 57.5±17.6 µM, respectively, Figure 2B), which is consistent with previous results [26]. However, SA levels were 4- to 5-fold lower in leaves that had been pre-treated with AtGV3101 for 24 or 48 h (Figures 2A and B; 48 h data shown). SA-spiking experiments in which ADPWH-*lux* was co-infiltrated with 100 µM SA confirmed that inhibition could not be explained in terms of a suppressive effect of AtGV3101 on biosensor activity (data not shown).

To examine whether the effect of AtGV3101 on SA elicitation by *P. syringae* was confined to the apoplastic pool of SA, we also examined SA levels in whole leaf extracts (Figure 2C). Although the values obtained from whole leaf samples are expressed as µg SA/g of freeze-dried tissue and thus cannot be directly compared to values obtained with the biosensor, there was a comparable reduction in SA in both apoplast and whole leaf samples (a 4- and 3-fold reduction in Pta- and Pto-challenged leaves, respectively), which suggests that the effect of AtGV3101 on *P. syringae*-induced SA is associated with an overall reduction in SA synthesis rather than apoplast-specific processes such as inhibition of SA transport or degradation of SA. Consistent with this, we were able to use ADPWH-*lux* to confirm that AtGV3101 did not deplete SA concentrations when incubated in synthetic medium or in *N. tabacum* apoplast extracts supplemented with 100 µM SA (Figures S1A and B).

It is well established that pathogen-induced accumulation of SA in *N. tabacum* leaves leads to the induction of pathogenesis-related (PR) proteins and the establishment of SAR [11]. As expected, both Pta and Pto elicited *PR1a* expression after 24 h in MgCl_2_(AS)-pre-treated leaves, but *PR1a* expression was reduced between 3- and 2-fold respectively in leaves that had been pre-treated with AtGV3101 and later inoculated with Pta and Pto, compared to the MgCl_2_(AS) pre-treatment (2.69±0.71 fold in leaves inoculated with Pta and 2.21±0.35 fold in leaves inoculated with Pto; average of three independent experiments ± standard error; see Figure 2D for a representative experiment). Collectively, the two independent measurements of SA in the apoplast and in whole leaves, and measurement of *PR1a* expression demonstrate that pre-treatment with AtGV3101 suppresses both SA synthesis and SA-mediated responses elicited by *P. syringae*.

### Pre-Treatment of Leaves with High Inoculum Densities of Living *A. tumefaciens* Is Needed to Obtain Maximum Suppression of *P. syringae-*Elicited SA Production

**Figure 3:**
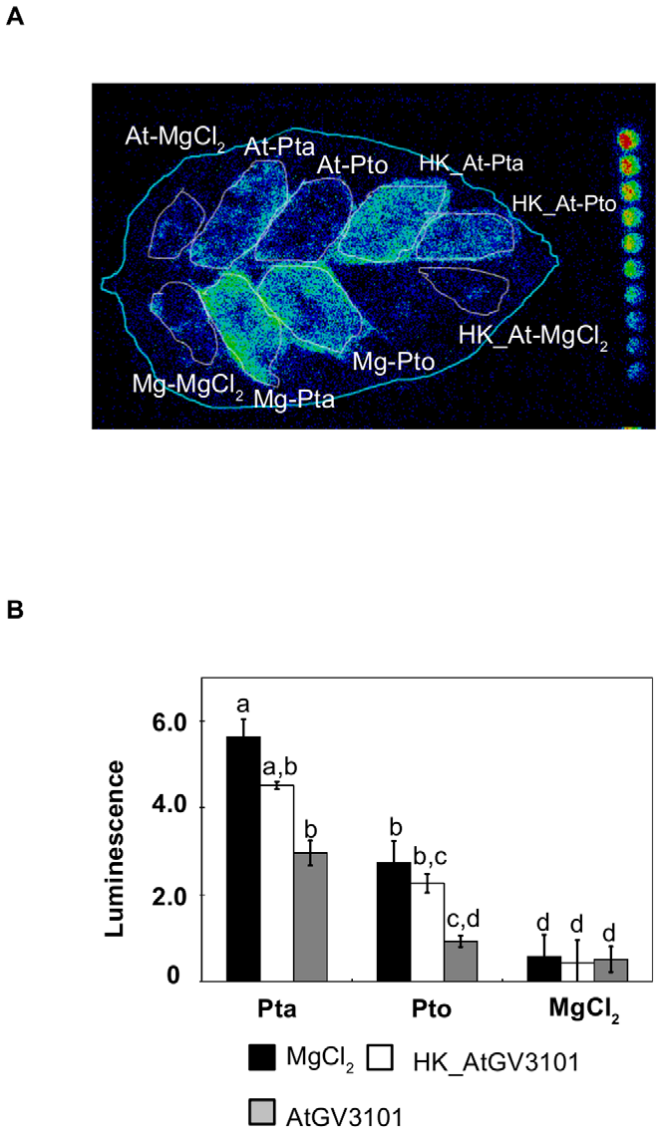
Heat-killed *A. tumefaciens* suppresses *P. syringae*-elicited SA production to a lesser extent than live *A. tumefaciens*. **A.** The leaf shown was inoculated with *A. tumefaciens* GV3101 (AtGV3101 10^7^ cfu/ml), heat-killed AtGV3101 (HK_At) or 10 mM MgCl_2_(AS) (Mg), followed by inoculation with *P. syringae* pv. tabaci 11528 (Pta), *P. s.* pv. tomato DC3000 (Pto) (10^5^ cfu/ml) or 10 mM MgCl_2_ after 48 hours. The SA biosensor ADPWH-*lux* was inoculated into leaves 24 hours after infiltration with *P. syringae*. SA-induced luminescence was measured one hour after biosensor inoculation using a photon-counting camera. **B.** Absolute lux values were normalized against the infiltrated area. The bars show the average normalized luminescence values from three leaves. Error bars show standard deviation. General Linear Model (GLM) analysis revealed statistical differences between treatments (F = 81.2673; p<0.0001; df = 26). Means with the same letter were not significantly different at the 5% confidence level based on Tukey's HSD Test.

Having shown that pre-treatment with AtGV3101 suppressed pathogen growth, symptom development, defence-associated cell death (HR) and SA production, we performed a series of experiments to define the conditions in which AtGV3101 was able to suppress *P. syringae*-elicited symptoms and SA production. Using the same experimental set up as described above, we observed that a suspension of heat-killed AtGV3101 retained some ability to suppress *P. syringae*-elicited SA, but was less able to suppress SA than live bacteria (Figure 3). This is in accordance with results published by Pruss and collaborators [17], which showed that heat-killed *A. tumefaciens* was less effective at protecting tobacco leaves from infection by TMV than live bacteria. Nevertheless, the observation that heat-killed bacteria retain some ability to suppress SA elicitation provides some support for the hypothesis that plant responses to *A. tumefaciens* PAMPs contribute to the effect of *A. tumefaciens* on *P. syringae-*plant interactions.

Minimum inoculation densities of 10^7^ and 10^6^ cfu/ml of AtGV3101 were needed to obtain consistent suppression of the SA production elicited by Pta and Pto respectively (Figure S2). However, co-inoculation of AtGV3101 at 10^7^ cfu/ml with *P. syringae* at 10^5^ cfu/ml failed to suppress SA production (Figure S3). This argues against the hypothesis that suppression is due to direct inhibition of *P. syringae* as a result of nutrient competition or antagonism, and in favour of the hypothesis that inoculation of high levels of AtGV3101 prior to inoculation with other pathogens elicits host responses that make plant tissues more resistant to subsequent infection.

### *A. tumefaciens* Does Not Suppress SA Production Elicited by HrpZ

**Figure 4:**
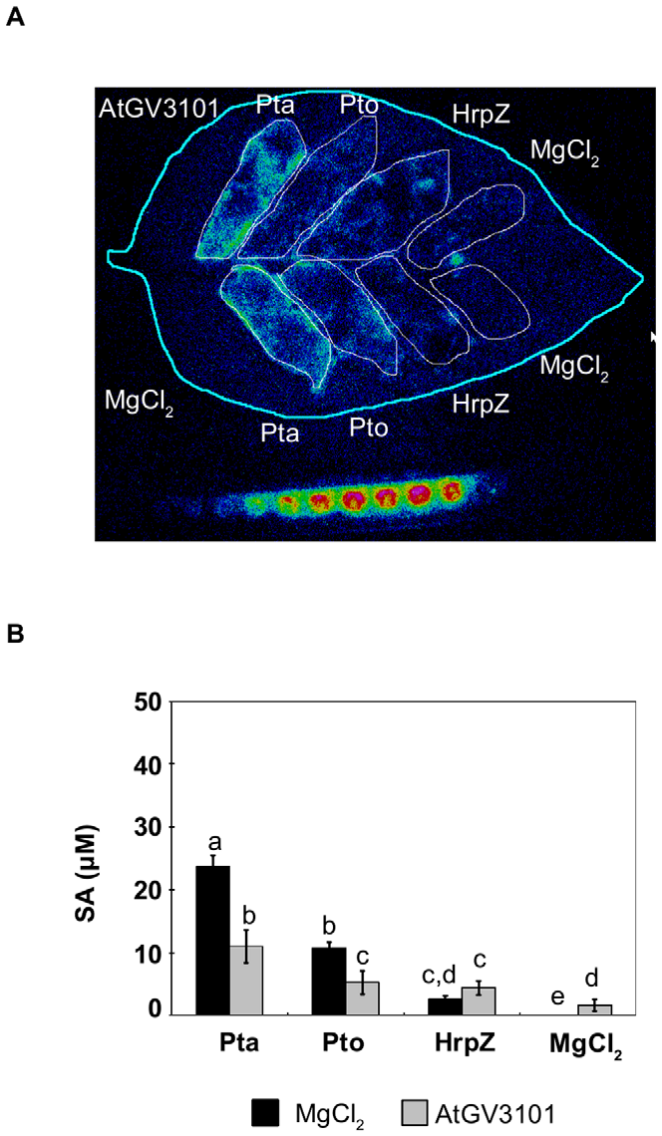
*A. tumefaciens* is unable to suppress HrpZ-elicited SA production. **A.** The leaf shown was inoculated with *A. tumefaciens* GV3101 (AtGV3101 10^7^ cfu/ml), or 10 mM MgCl_2_(AS) (Mg), followed by inoculation with *P. syringae* pv. tabaci 11528 (Pta), *P. s.* pv. tomato DC3000 (Pto) (10^5^ cfu/ml), HrpZ (0.1 mg/ml) or 10 mM MgCl_2_ after 48 hours. The SA biosensor ADPWH-*lux* was inoculated into leaves 24 hours after infiltration with *P. syringae* or HrpZ. SA-induced luminescence was measured one hour after biosensor inoculation using a photon-counting camera. **B.** Numerical SA values were calculated using a calibration curve as described in Huang et al. [26]. The bars show the average SA values from three independent experiments. Bars show standard error of the mean. General Linear Model (GLM) analysis revealed statistical differences between treatments (F = 22.4936; p<0.0001; df = 23). Means with the same letter were not significantly different at the 5% confidence level based on Student's t test.

The observation that AtGV3101 suppressed both the HR and SA production could indicate that AtGV3101 has a repressive effect on plant signal transduction pathways leading to defence-associated cell death. We used the type III secreted protein, HrpZ, which has been shown to elicit both SA production and the HR in *N. tabacum* [27], to examine the effect of AtGV3101 in the absence of AtGV3101-*P. syringae* interactions. As expected, at 1 mg/ml, HrpZ elicited a macroscopic HR (not shown). The tissue collapse associated with the macroscopic HR prevents infiltration of the SA biosensor, so we infiltrated HrpZ at 0.1 mg/ml to observe the SA response. AtGV3101 did not have a significant effect on the amount of necrosis elicited by 1 mg/ml HrpZ, or on the amount of SA elicited by 0.1 mg/ml HrpZ (2.623±0.506 µM (MgCl_2_) and 4.415±1.127 µM (AtGV3101); 3 replicates, p value>F = 0.1783; df = 5) (Figure 4). However, SA production in response to 0.1 mg/ml HrpZ was notably lower than SA production in response to Pto or Pta. These results suggest that the effect of AtGV3101 on the *P. syringae-*elicited HR and on *P. syringae*-elicited SA production involves pathways or mechanisms that are not active during plant responses to HrpZ.

### Suppression of *P. syringae*-Elicited SA Production by *A. tumefaciens* Is Not Dependent on the Type IV Secretion System

**Figure 5:**
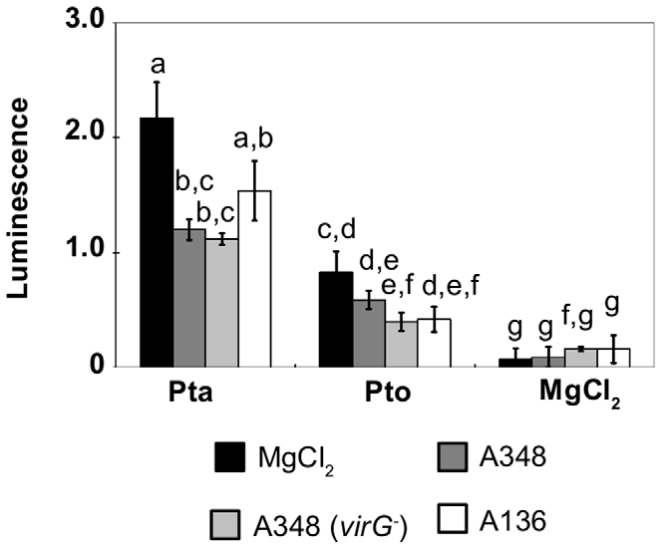
*A. tumefaciens* strains lacking *virG* and pTi are able to suppress SA production in *N. tabacum*. Tobacco leaves were inoculated with wild type *A. tumefaciens* A348 (dark grey bars), A348 (*virG*-) (pale grey bars), the pTi lacking derivative A136 (white bars) (all at 10^7^ cfu/ml) or 10 mM MgCl_2_(AS) (black bars), followed by inoculation with *P. syringae* pv. tabaci 11528 (Pta) or *P. s.* pv. tomato DC3000 (Pto) (10^5^ cfu/ml) after 48 hours. The SA biosensor ADPWH-*lux* was inoculated into leaves 24 hours after infiltration with *P. syringae*. SA-induced luminescence was measured one hour after biosensor inoculation using a photon-counting camera and absolute lux values were normalized against the infiltrated area. Error bars show standard error of the mean. General Linear Model (GLM) analysis revealed statistical differences between treatments (F = 21.0735; p<0.0001; df = 50). Means with the same letter were not significantly different at the 5% confidence level based on least square (LS) means using Student's *t-*test. The experiment was performed at least twice with similar results.

AtGV3101 is a non-oncogenic derivative of *A tumefaciens* C58 that carries the plasmid pMP90, which lacks the T-DNA region but contains the *vir* genes that encode the type IV secretion system [28]. To investigate whether the type IV secretion system or other Ti plasmid-borne genes contribute to the mechanism by which AtGV3101 suppresses *P. syringae*-elicited SA production, we tested three derivatives of *A. tumefaciens* C58 using the SA biosensor assay described earlier: *A. tumefaciens* A136 is a derivative of C58 cured of its octopine Ti plasmid and carrying the single cryptic plasmid pATC58 [29], *A. tumefaciens* A348 is isogenic with A136, and carries the Ti plasmid pTiA6 [30], *A. tumefaciens* A348 (*virG* ^−^) lacks a functional copy of *virG*, which encodes a positive regulator of type IV protein secretion [31]. All three strains displayed a similar ability to suppress SA synthesis induced by Pta and Pto, although in some experiments, the plasmid-cured strain A136 was marginally less effective at suppressing SA synthesis in response to Pta (Figure 5). This is consistent with the observation that a plasmid-cured strain of *A. tumefaciens* was less able to suppress infection by TMV [17], and suggests that plasmid-borne genes play a minor, but non-essential role in SA suppression.

### Infiltration of *A. tumefaciens* into *N. tabacum* Leaves Results in Reduced ABA Levels and Primes Callose Deposition

**Figure 6:**
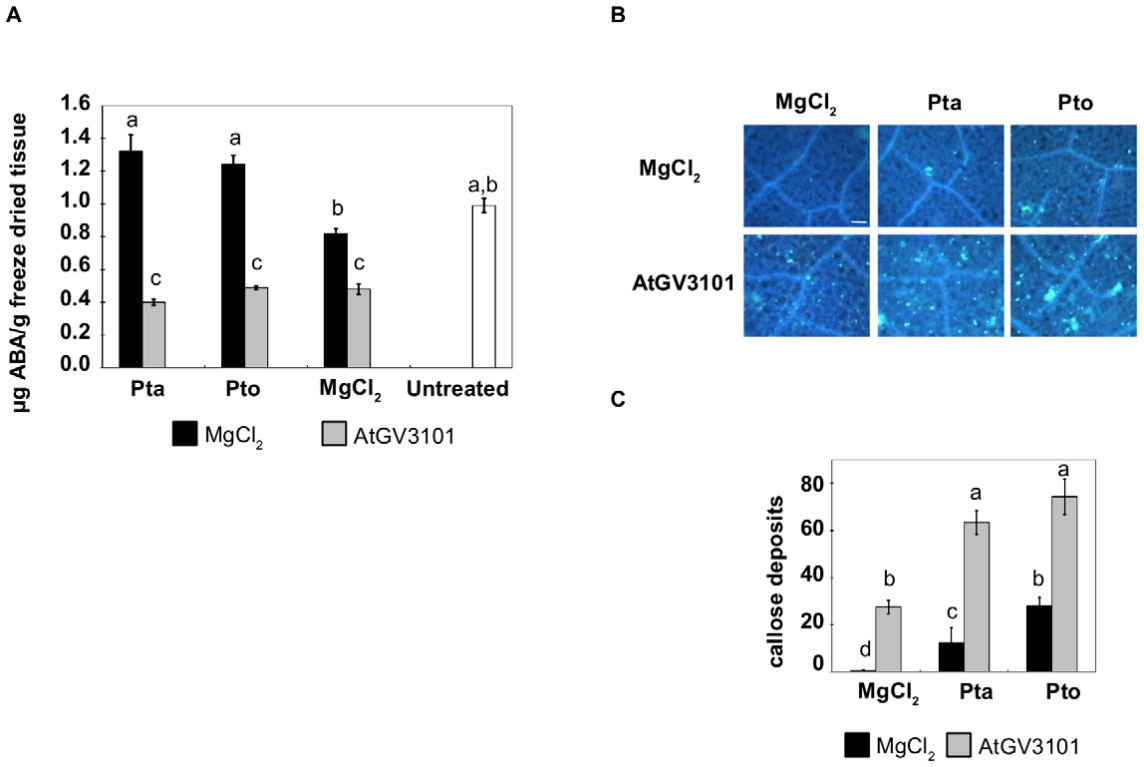
Agroinfiltration decreases ABA levels and primes callose deposition in *N. tabacum*. Leaves were inoculated with *A. tumefaciens* GV3101 (AtGV3101) at 10^7^ cfu/ml or with 10 mM MgCl_2_(AS), followed 48 hours later by *P. syringae* pv. tabaci 11528 (Pta) or *P. s.* pv. tomato DC3000 (Pto) at 10^5^ cfu/ml, or 10 mM MgCl_2_. **A.** ABA levels were determined for whole leaf samples by LC/MS/MS. The graphs show average values from five independent experiments. The bars indicate the standard error of the mean. One way ANOVA revealed statistical differences between treatments (F = 11.1058; p<0.0001; df = 6). Means with the same letter were not significantly different at the 5% confidence level based on Student's t-test. **B.** Leaf sections were excised 24 hours after infiltration with *P. syringae*, stained with aqueous aniline blue, and imaged under ultraviolet excitation at 370 nm. Pictures are representative of five areas of 1.3 mm^2^ taken from a leaf section. Two sections from two leaves were stained in each experiment and the experiment was performed twice with similar results. Scale bar, 100 µm. **C.** Average callose deposits per field of view (1.3 mm^2^). The bars indicate the standard error of the mean. GLM revealed statistical differences between treatments (F = 58.7652; p<0.0001; df = 5). Means with the same letter were not significantly different at the 5% confidence level based on Tukey's HSD Test.

One mechanism by which pathogens suppress plant defence responses is by manipulation of plant hormones [32]. Both the wound and developmental signal JA and the abiotic stress hormone ABA have been implicated in suppression of plant defences during *P. syringae* infection [32]–[36]. However, relatively little is known regarding the effect that *A. tumefaciens* has on ABA or JA levels in plants. To assess whether pre-treatment with AtGV3101 modified hormone levels in *N. tabacum* we measured both ABA and JA in the experimental conditions described here and found that, as expected, inoculation of MgCl_2_(AS)-pre-treated plants with Pto or Pta resulted in a significant increase in ABA. However, we observed a marked decrease in ABA in leaves that were pre-treated with AtGV3101, to the extent that ABA levels in AtGV3101-treated leaves were lower than in untreated control leaves (Figure 6A). Interestingly, we did not observe detectable amounts of JA in leaves challenged with Pta or Pto (data not shown), in contrast to the increased JA levels previously reported in Pto-infected *Arabidopsis* leaves [32]. This is consistent with a previous study by Huang and collaborators [37], who reported that *N. tabacum* plants inoculated with Pto or Pta produced the volatile SA derivative MeSA, but found that neither Pto or Pta elicited significant production of JA.

The observation that *P. syringae* grew to lower population densities in AtGV3101 pre-treated leaves compared to control leaves suggested that although AtGV3101 suppressed some defence responses, other defences were enhanced by the presence of AtGV3101. SA-independent defence responses such as callose deposition have been shown to exhibit a strong correlation with plant resistance to *P. syringae* [38], so we examined the number of callose deposits in infected and healthy *N. tabacum* leaves pre-treated with AtGV3101 or MgCl_2_(AS)_._ Neither Pto nor Pta elicited callose deposition in MgCl_2_(AS)-treated *N. tabacum* leaves, which indicates that basal defences such as callose deposition were effectively suppressed by the pathogenicity and virulence factors produced by these two strains of *P. syringae* [39], [40]. However, callose deposits were observed in leaves pre-treated with AtGV3101, and were present in high numbers in Pto- and Pta-infected leaves that had been pre-treated with AtGV3101 (Fig. 6B and C). This indicates that pre-treatment of *N. tabacum* with AtGV3101 primes callose deposition, or reduces the ability of Pto and Pta to suppress the basal immune response.

## Discussion

The observation that infiltration of *N. tabacum* leaves with AtGV3101 reduced the symptoms caused by *P. syringae* prompted us to investigate the mechanistic basis of this phenomenon. Our results show that at inoculum densities typically used for transient expression, AtGV3101 reduces ABA levels, elicits low levels of callose deposition and alters plant responses to virulent and non-host strains of *P. syringae*, suppressing necrosis, SA synthesis and *PR1a* expression, but increasing callose deposition.

**Figure 7:**
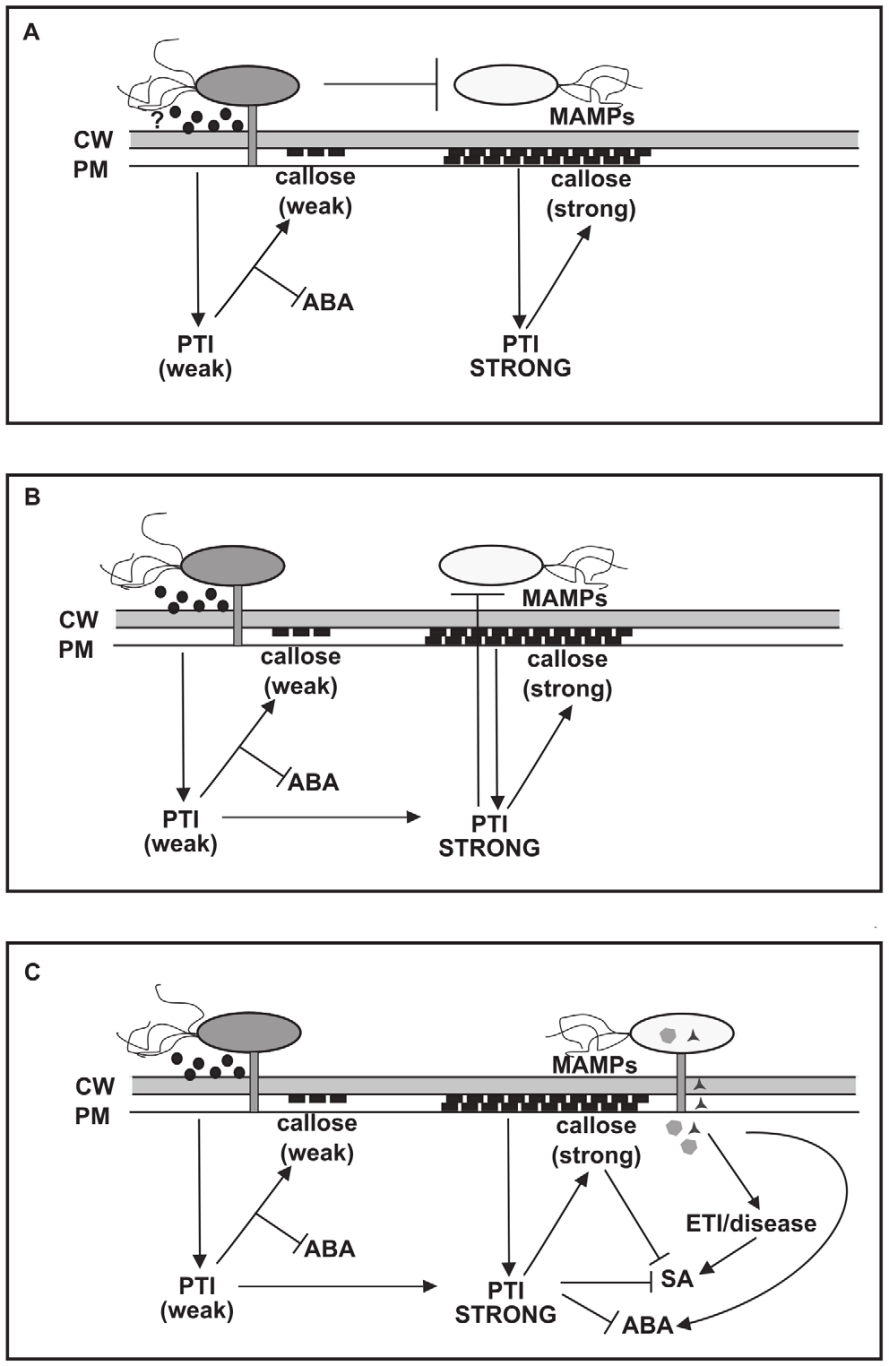
Potential mechanisms underlying the effect of *A. tumefaciens* on *P. syringae-*plant interactions. (A) *A. tumefaciens* inhibits proliferation or virulence gene expression in *P. syringae*. The presence of high densities of *A. tumefaciens* in the plant apoplast could have a direct inhibitory effect on proliferation and virulence gene expression in *P. syringae*. This, in turn, could enhance PAMP-triggered immunity (PTI) elicited by *P. syringae* MAMPS and suppress SA production. Infiltration of AtGV3101 also enhances some basal defence responses and ABA levels are reduced. (B) *A. tumefaciens-*mediated priming of the basal immune response inhibits proliferation or virulence gene expression in *P. syringae*. The observation that heat-killed AtGV3101 partially suppresses *P. syringae*-elicited SA argues against a direct interaction between AtGV3101 and *P. syringae* as the sole cause for SA suppression and enhanced expression of basal defences. An alternative explanation would be that AtGV3101 primes for an enhanced basal immune response, which has a negative effect on the ability of *P. syringae* to suppress PTI, and on the ability of *P. syringae* to elicit salicylic acid (SA) synthesis as a result of effector-triggered immunity (ETI) or disease. (C) *A. tumefaciens-*mediated priming of the basal immune response suppresses SA and ABA synthesis. In addition to inhibiting the activities of *P. syringae*, an enhanced basal immune response could directly suppress both SA and ABA synthesis. CW: cell wall; PM: plasma membrane; ABA: abscisic acid; MAMPs: microbe-associated molecular patterns.

This is not the first study to report that *A. tumefaciens* modifies plant responses to *P. syringae*, as Robinette and Matthysse [41] have previously reported that pre-treatment with wild type *A. tumefaciens* C58 eliminated the HR elicited by *Pseudomonas syringae* pv. phaseolicola (Pph) in *N. tabacum*. However, they concluded that *vir-*independent auxin biosynthesis by the products of genes located in the T-DNA region of the Ti plasmid was required for HR inhibition. In our experiments, a disarmed strain of *A. tumefaciens* that lacked the ability to transform plant cells with auxin biosynthesis genes suppressed *P. syringae-*induced SA synthesis when inoculated at densities typically used for transient gene expression, and similar results were obtained with strains that lacked the Ti plasmid or carried a mutation in *virG*. This demonstrates that Ti-plasmid dependent auxin synthesis is not required for *A. tumefaciens* to modify plant responses to *P. syringae*, and suggests that the results described in this paper illustrate a mechanistically distinct phenomenon. There are clear parallels between our work and that of Pruss and collaborators [17], who showed that agroinfiltration of tobacco leaves with disarmed strains of *A. tumefaciens* resulted in increased resistance to TMV and SA-independent chlorosis and inhibition of leaf expansion over several days. Interestingly Pruss *et al.* also showed that agroinfiltration of wild-type *A. tumefaciens* elicited a T-DNA dependent increase in miR393, which has been linked to PAMP-dependent repression of auxin signalling, suggesting that T-DNA-dependent effects on plant defence responses, such as those reported by Robinette and Matthysse [41], could be linked to either increased or decreased auxin, depending on the stage of infection. The possible mechanisms by which disarmed strains of *A. tumefaciens* alter plant interactions with *P. syringae* are outlined in Figure 7 and discussed below.

First, it is possible that the presence of high densities of *A. tumefaciens* in the plant apoplast has a direct effect on the proliferation or virulence of *P. syringae* by occupying the intercellular space, producing anti-microbial chemicals and extracellular signalling molecules and competing with *P. syringae* for nutrients, all of which could reduce the ability of *P. syringae* to grow and induce host responses (Figure 7a). However, AtGV3101 was unable to suppress *P. syringae* plant-interactions when co-infiltrated with *P. syringae* (data not shown), and we observed detectable inhibition even when using heat-killed cells (Figure 3), which indicates that *A. tumefaciens* does not need to be alive to exert an inhibitory effect. This argues against direct antagonism and competition as the sole reason for the effect of AtGV3101 on interactions between *N. tabacum* and *P. syringae*. Nevertheless, as the highest level of inhibition was observed with living *A. tumefaciens* cells containing a disarmed Ti plasmid, it seems likely that *A. tumefaciens* needs to become established in plant tissues and to express plant-induced genes before it can most effectively interfere with *P. syringae* growth and *P. syringae*-plant interactions.

An alternative explanation for the effect of AtGV3101 on plant interactions with *P. syringae* is that it could be priming basal defence responses (Figure 7b,c). We observed that at concentrations normally used for transient expression assays, AtGV3101 caused a significant decrease in ABA levels and elicited moderate callose deposition, and when plants were subsequently inoculated with *P. syringae*, we observed reduced SA synthesis and high levels of callose deposition. The hypothesis that *A. tumefaciens* primes plant defences is consistent with reports that signal transduction, gene expression and defence responses are altered upon infection of *N. tabacum* by non-oncogenic *A. tumefaciens* [3], [17], [18], and that pre-treatment with non-oncogenic *A. tumefaciens* induces plant resistance to TMV, a phenomenon in which direct interactions between apoplastic *A. tumefaciens* and cytoplasmic TMV are unlikely to affect the outcome of pathogen infection [17]. However, in their study, Pruss and collaborators [17] observed a significant increase in *PR1a* expression in response to *A. tumefaciens* ASE/pPZP211, while in our experiments *A. tumefaciens* treatment alone did not alter SA production and *PR1a* expression. This discrepancy may be linked to the fact that they used a significantly higher density of *A. tumefaciens* (8×10^8^ cfu/ml as opposed to 1×10^7^ cfu/ml), which could have had a more pronounced impact on *PR1a* expression. Pruss and collaborators [17] speculated that stimulation of SA-dependent defences by *A. tumefaciens* could contribute to increased resistance to TMV, but in our system this seems less likely.

The identity of the *A. tumefaciens* factor or factors that prime callose synthesis and suppress *P. syringae*-elicited SA responses in *N. tabacum* remains unclear, but the observation that some suppression was observed with heat-killed cells indicates that one or more MAMPs present in heat-killed bacteria contribute to priming and SA suppression by triggering PTI. SA suppression occurred in response to both a *virG^−^* mutant and a Ti-plasmid-cured strain of *A. tumefaciens* C58, which indicates that at least one trait involved in this phenomenon is encoded by a chromosomal, Vir-independent gene. Robinette and Matthysse [41] observed that *A. tumefaciens* strains carrying a mutation in the chromosomal β-D-1,2-glucan synthesis gene *chvB*, which disables the ability of bacteria to attach to plant cells and produce high levels of extracellular polysaccharide showed a reduced ability to inhibit a Pph-elicited HR. This suggests that recognition of *A. tumefaciens* by plant cells benefits from close contact between host cells and bacteria, and raises the possibility that defences are elicited by surface-associated MAMPs.

As shown in Figure 7c, it is possible that callose production in response to *A. tumefaciens* MAMPs could result in callose-dependent suppression of SA synthesis in response to *P. syringae* [42], [43]. Alternatively, the enhanced basal defences observed in response to *A. tumefaciens* could have a negative effect on the ability of *P. syringae* to express or use virulence factors to suppress basal defences, and to proliferate in plant tissues (Figure 7b). For example, Oh and Collmer [44] showed that the HR elicited by Pto DC3000 was suppressed in *N. benthamiana* leaves that had been primed for basal defences. Furthermore, Klement and collaborators [45] demonstrated that induction of local induced resistance (IR) prior to infiltration of *P. syringae* can suppress expression of the *hrp-*encoded type III secretion system (TTSS), which is known to be involved in both pathogenesis and HR elicitation.

Interestingly, when we used HrpZ instead of Pto to elicit the HR, we did not observe a decrease in SA accumulation or necrosis (Figure 4 and data not shown). This suggests that HrpZ is able to by-pass any effect of *A. tumefaciens* on plant cells. Analyses of the signal transduction mechanisms involved in HR elicitation by HrpZ have shown that the HR elicited by HrpZ is SA-dependent, while the *P. syringae* HR is potentiated by, but is not dependent on SA [27], [46]–[48]. It is therefore conceivable that the increase in SA synthesis elicited by HrpZ and *P. syringae* involves distinct pathways, and that the HrpZ-dependent pathway is not subject to interference arising from pre-treatment with *A. tumefaciens*. Alternatively, our results could indicate that the low level of PTI elicited by AtGV3101 is not sufficient to have a suppressive effect on HrpZ-induced SA synthesis and that suppression only occurs in response to a stronger *P. syringae*-dependent PTI response observed after treatment with *A. tumefaciens* and *P. syringae*.

The reduction in ABA levels observed in *N. tabacum* leaves after infiltration of *A. tumefaciens* (Figure 6A) poses interesting questions. The role of ABA in plant-pathogen interactions is complex and dependent on the pathogen's lifestyle [32], [42], [49]. Several studies have shown that increased ABA levels can contribute to increased susceptibility to biotrophic pathogens [21], [50], [51] and *P. syringae* has been shown to promote increased ABA levels in *Arabidopsis* leaves. This increase in ABA levels aids the infection process by suppressing basal defences [32], [34]. As expected, we observed increased ABA levels in mock inoculated *N. tabacum* leaves that were later inoculated with *P. syringae*, along with little or no callose deposition. The role of ABA in suppressing basal defences in *Arabidopsis* is known to include the attenuation of callose deposits and suppression of PAMP-induced genes (PIGs) [32], [52]. It is therefore conceivable that the reduction in ABA levels in agroinfiltrated leaves could contribute to priming of plant defences and enhanced callose deposition (Figure 6B and C). In support of this, Asselbergh and collaborators [53] have recently reported that the ABA-deficient tomato mutant *Solanum lycopersicum sitiens* shows an enhanced basal immune response to the soft-rot pathogen *Dickeya dadantii (Erwinia chrysanthemi*).

We were unable to detect significant levels of JA in any of our treatments, which is consistent with a previous study by Huang and collaborators [37], and suggests that JA plays a relatively minor role in the hormonal interplay that occurs during *A. tumefaciens* and *P. syringae* interactions with *N. tabacum*. This contrasts with the central role of JA signalling in *P. syringae-*tomato and *P. syringae-Arabidopsis* interactions [54]–[56]. We did not examine the impact of disarmed strains of *A. tumefaciens* on auxin, which might also be expected to have a significant impact on plant defence responses if altered, and to suppress SA signalling if increased [21]. However, as noted above, both this study and previous work by Pruss and collaborators [17] have shown that the ability of *A. tumefaciens* to suppress pathogen-induced necrosis and SA synthesis is independent of T-DNA dependent auxin synthesis and T-DNA dependent elicitation of the auxin-repressing miRNA miR393. Nevertheless, it would be interesting to examine whether miR393 or auxin levels are altered in agroinfiltrated leaves following inoculation of *P. syringae*, as increased expression of miR393 might be expected to occur in conjunction with enhanced expression of basal defence responses.

### Concluding Remarks

Collectively, our results show that infiltration of *N. tabacum* leaves with AtGV3101 prior to challenge with *P. syringae* induces physiological and regulatory changes in plant cells, and possibly in *P. syringae*, which result in increased basal defences and restriction of pathogen growth. Further studies are needed to confirm the identity of the factor, or factors that prime plant defences during *A. tumefaciens-N. tabacum* interactions, and to assess whether *A. tumefaciens* has a direct or plant-mediated effect on pathogen growth, behaviour and expression of pathogenicity factors.

*A. tumefaciens*-mediated transient expression has many advantages over conventional transformation approaches for high-throughput studies of gene function, and remains an attractive system for a wide variety of applications. However, the results described in this paper clearly show that data obtained using *A. tumefaciens*-mediated transient expression to study plant-pathogen interactions or hormone-mediated signal transduction should be interpreted with caution. Stably transformed plants, and transient expression approaches based on virus-mediated gene expression may provide more accurate information on cellular processes associated with plant defence responses and plant hormone-signalling than agroinfiltration.

## Materials and Methods

### Plant Material and Bacterial Strains

Tobacco (*N. tabacum* L. cv Xanthi) seeds were sown in a 3∶1 soil: vermiculite mix and grown in a cooled incubator (LMS Ltd, Kent, UK) under a 16 h photoperiod and 21°C temperature. When cotyledons were visible, seedlings were transferred to 1 l pots and grown under the same conditions for 6 to 8 weeks.

The bacterial strains used were: *P. syringae* pv. tomato DC3000 [57], *P. syringae* pv. tabaci ATCC11528 (American type culture collection), *Acinetobacter baylyi* ADPWH_*lux* [58], and the *A. tumefaciens* C58 derivatives *A. tumefaciens* GV3101 (pMP90) [28], *A. tumefaciens* A136, an avirulent derivative which lacks the tumor inducing plasmid (pTi) [29], *A. tumefaciens* A348, which is isogenic with strain A136 and harbours the pTi plasmid pTiA6NC [29] and *A. tumefaciens* MX19, which has an insertional mutation in *virG* [31]. All strains were routinely cultured at 28°C on Luria-Bertani (LB) agar or in LB broth [59] and antibiotics were added when appropriate (Gentamicin 20 µg/ml; Nitrofurantoin 50 µg/ml).

### Plant Inoculation and Bacterial Growth *In Planta*

To examine the effect of AtGV3101 on the growth of *P. syringae in planta* and on plant defence responses to *P. syringae*, an overnight liquid culture of live or heat-killed AtGV3101 was re-suspended in 10 mM MgCl_2_ with 0.5 mM acetosyringone (MgCl_2_(AS)) at approximately 10^7^ cfu/ml and infiltrated into leaves. Ten millimolar MgCl_2_(AS) was used as a negative control. Plants were incubated in the growth conditions described above, and after 48 h, overnight cultures of Pta and Pto were re-suspended in 10 mM MgCl_2_ and infiltrated at 10^5^ cfu/ml. For SA biosensor assays leaves were infiltrated with the SA biosensor ADPWH_*lux* 24 h after inoculation of Pta and Pto, as discussed below. For HR assays Pta and Pto were infiltrated at 10^7^ cfu/ml. Experiments were also carried out in which leaves were co-inoculated with AtGV3101 and *P. syringae*, or pre-treated with AtGV3101 for 24 h prior to inoculation with *P. syringae*, in order to examine whether pre-treatment with AtGV3101 was necessary for modulation of plant defences. To heat-kill *A. tumefaciens*, the resuspended cells were heated at 70°C for 30 min, then cooled at room temperature and diluted to 10^7^ cfu/ml. No colonies were recovered after plating 100 µl of heat-treated cells. HrpZ was prepared and purified as described by Lee and collaborators [60]. Crude protein extracts of 1 mg/ml and 0.1 mg/ml were used to infiltrate tobacco leaves.

For population counts each replicate consisted of two leaf discs of 0.3 cm^2^, which were collected from leaves at 0, 3, 5 and 11 days after inoculation of *P. syringae*. Leaf discs were homogenised in 10 mM MgCl_2_ and the resulting suspension was dilution plated on LB agar supplemented with Gentamicin (10 µg/ml) for selection of AtGV3101 and Nitrofurantoin (50 µg/ml) for selection of *P. syringae* strains and incubated at 28°C overnight.

### Salicylate Utilisation Assays

An overnight culture of AtGV3101 was resuspended at OD_600_ 0.02 in 100 µl of LB, M9 minimal medium [59], or *N. tabacum* apoplast extract [61], or in media and apoplast extracts supplemented with 100 µM SA, and incubated for 24 h in a 96-well plate at 28°C with shaking. Media and apoplast extracts supplemented with SA were used as internal standards. AtGV3101 growth was measured after 24 h at OD_600_ using a Tecan Infinite® 2000 plate reader (Tecan Ltd, Männedorf, CH). To measure SA levels, the plate was centrifuged at 3000 rpm for 5 min and 20 µl of supernatant were mixed with 50 µl of *A. baylyi* ADPWH_*lux* (OD_600_ 0.4) and 30 µl of LB in a black clear-bottom 96-well plate. Bioluminescence and OD_600_ were measured after incubation for 1 hour at 28°C with shaking. Relative bioluminescence was obtained by dividing bioluminescence by OD_600_ to estimate the SA concentration as described previously [26], [58].

### Use of *A. baylyi* ADPWH_*lux* to Detect SA *In Planta*

*A. baylyi* ADPWH_*lux* (ADPWH_*lux*) was used to detect SA *in planta* as described by Huang and collaborators [26] with slight modifications. To prepare bacteria for infiltration, a single colony of ADPWH-*lux* was inoculated into 5 ml of LB broth and incubated with shaking overnight at 28°C. The overnight culture was diluted into 100 ml of LB and incubated with shaking at 37°C for approximately 2 h to reach OD_600_ 0.4, at which point aliquots of the ADPWH_*lux* culture were infiltrated into leaves using a blunt 1 ml syringe. Plants were maintained at 21°C for one hour prior to imaging. SA spiking experiments were performed to confirm that treatments did not interfere with the activity of the biosensor by mixing 100 µM SA with the biosensor immediately prior to inoculation.

### Imaging and Quantification of Bacterial Luminescence *In Planta*

SA-induced luminescence was imaged in the dark using a photon-counting camera (Photek Ltd., East Sussex, UK) one hour after infiltration of the ADPWH_*lux* biosensor. Leaves were acclimated to the dark for at least 100 seconds to allow photosynthesis-associated autoluminescence to disappear, and then luminescence counts were recorded every second for 200 seconds. *In vitro* SA concentration ladders were included with each imaged leaf to allow comparison of bioluminescence between separate images and were prepared as described previously [26] to yield final SA concentrations of 0, 0.02, 0.05, 0.1, 0.2, 0.3, 0.4, 0.5, 1.0 and 2.0 µM in a final volume of 100 µl. The total pixel intensity for each inoculated area was obtained using Photek IFS32 image processing and data acquisition software (Photek Ltd.) and normalized luminescence values were obtained by dividing the total counts by number of pixels in the inoculated area. Numerical SA values were calculated using a calibration curve as described in [26]. Briefly, *N. tabacum* leaves were used to produce *in vivo* SA concentration ladders by mixing various concentrations of SA with the biosensor prior to infiltration into the leaf. Images of the *in vivo* SA concentration ladders from at least two separate leaves were used to construct a calibration curve.

### Measurement of SA, ABA and JA in *N. tabacum* Leaf Extracts by LC/MS/MS

To confirm the SA measurements obtained with ADPWH_*lux* and to obtain whole leaf data for SA, ABA and JA, plants were inoculated with AtGV3101 (10^7^ cfu/ml) and *P. syringae* (10^5^ cfu/ml) as described above. Twenty four hours after inoculation of *P. syringae*, plant material was harvested in liquid nitrogen and freeze-dried. To obtain homogeneous samples, samples from three leaves of 6 to 8 week-old *N. tabacum* plants were pooled for each treatment. Ten milligrams of freeze-dried tissue were processed for each sample and contents of SA, ABA and JA levels were analysed as described previously [62].

### Analysis of *PR1a* Defense Gene Expression

To study the effect of AtGV3101 on SA-dependent gene expression, plants were inoculated with AtGV3101 (10^7^ cfu/ml) and *P. syringae* (10^5^ cfu/ml) as described above, and expression was analysed 24 h after inoculation of *P. syringae* by quantitative real-time RT-PCR (qRT-PCR), using *EF-1*α and *actin* as internal controls. Leaf samples were homogenised in liquid nitrogen and RNA was isolated using the RNeasy® Plant Mini kit (Qiagen GmbH, Hilden, Germany) according to the manufacturer's instructions. RNA was treated with RNAse free DNAse (Applied Biosystems/Ambion, Austin, USA) and random primed first-strand cDNA was generated using the ImProm-II™ Reverse Transcripton System (Promega Corp., Madison, WI, USA). The primers used to detect the relative abundance of *PR1a* were PR1aF (5′-GCGCAAAATTATGCTTCCCA-3′), PR1aR (5′-CCGTCATGAAATCGCCACTT-3′), EF1αF (5′-TCTGTTGAGATGCACCACGAAG-3′), EF1αR (ACAAACCCACGCTTGAGATCC-3′), NtActinF (5′-CCAGTGGCCGTACAACAGGTAT-3′) and NtActinR (5′-CCAACCGAAGAATTGCATGAG-3′). Real-time RT-PCR was performed with the SYBR Green® PCR Master Mix kit (Applied Biosystems). Each reaction was performed in triplicate plus a negative control using a 7300 Real Time PCR system (Applied Biosystems) and three independent experiments were performed. The comparative CT method [63] was used to quantify the relative expression ratio of *PR1a* in leaves infected with *P. syringae* that had been pre-treated with AtGV3101 or MgCl_2_.

### Callose Staining

To assess basal defence responses, leaves were inoculated with AtGV3101 (10^7^ cfu/ml) and *P. syringae* (10^5^ cfu/ml) as described above and leaf sections were excised 24 hours after infiltration with *P. syringae* and stained with aqueous aniline blue [64]. Leaf sections were imaged under ultraviolet excitation at 370 nm using an Olympus BX50 epifluorescence microscope. Two sections from two leaves were stained in each experiment and the experiment was performed twice.

## Supporting Information

Figure S1 *A. tumefaciens* does not degrade SA. A. The ability of *A. tumefaciens* AtGV3101 (AtGV3101) to degrade SA was examined in plant extracts and synthetic media. AtGV3101 was inoculated into LB or tobacco apoplast extracts (APOPLAST) supplemented with 100 µM SA. After 24 h of growth, 20 µl of the supernatant of the AtGV3101 cultures was mixed with 50 µl of the SA biosensor ADPWH-*lux* (grey bars) and SA-induced luminescence was measured after 1 hour. As a control, the SA biosensor was mixed with uninoculated media containing 100 µM SA (black bars). General Linear Model (GLM) analysis did not reveal statistical differences between treatments (F = 0.7589; p = 0.5479; df = 11). The experiment was performed twice with similar results. B. The inability of *A. tumefaciens* AtGV3101 to degrade SA was not due to inhibition of bacterial growth by SA or plant extracts. *A. tumefaciens* AtGV3101 was inoculated into LB, tobacco apoplast extracts and the same media supplemented with 100 µM SA. Growth was assessed after 24 hours. General Linear Model (GLM) analysis revealed statistical differences between treatments (F = 1384.993; p<0.0001; df = 11). Means with the same letter were not significantly different at the 5% confidence level based on Tukey's Honestly Significant Mean Differences (HSD) Test. Error bars in A and B show standard error of the mean.
(0.09 MB TIF)

Figure S2 High densities of *A. tumefaciens* are needed to suppress *P. syringae*-elicited SA. Tobacco leaves were inoculated with *A. tumefaciens* GV3101 (AtGV3101) at 10^7^, 10^6^, 10^5^ and 10^4^ cfu/ml or 10 mM MgCl_2_(AS) (MgCl_2_) followed by inoculation with *P. syringae* pv. tabaci 11528 (Pta) or *P. s.* pv. tomato DC3000 (Pto) (10^5^ cfu/ml) after 48 hours. The SA biosensor ADPWH-*lux* was inoculated into leaves 24 hours after infiltration with *P. syringae*. SA-induced luminescence was measured one hour after biosensor inoculation using a photon-counting camera and numerical SA values were calculated using a calibration curve as described in Huang et al. [26]. The chart shows average SA values from at least three leaves from different plants. Bars show standard error of the mean.
(0.08 MB TIF)

Figure S3 Co-inoculation of *A. tumefaciens* with *P. syringae* does not suppress *P. syringae*-elicited SA. Luminescence was examined 24 h after co-inoculation of 10^7^ cfu/ml AtGV3101 with 10^5^ cfu/ml Pta or Pto by infiltrating the ADPWH-*lux* SA biosensor in the inoculated zones. SA-induced luminescence was measured as described in Figure 2. The Y axis is shown in log scale. General Linear Model (GLM) analysis did not reveal statistical differences between treatments (F = 0.4547; p = 0.7283; df = 7). The experiment was performed twice with similar results. Error bars show standard deviation.
(0.04 MB TIF)

## References

[1] Chilton MD, Drummond MH, Merio DJ, Sciaky D, Montoya AL (1977). Stable incorporation of plasmid DNA into higher plant cells: the molecular basis of crown gall tumorigenesis.. Cell.

[2] Van Larebeke N, Engler G, Holsters M, Van den Elsacker S, Zaenen I (1974). Large plasmid in *Agrobacterium tumefaciens* essential for crown gall-inducing ability.. Nature.

[3] Ditt RF, Nester EW, Comai L (2001). Plant gene expression response to *Agrobacterium tumefaciens*.. Proc Natl Acad Sci USA.

[4] Hellens R, Mullineaux P, Klee H (2000). A guide to *Agrobacterium* binary Ti vectors.. Trends Plant Sci.

[5] Vaquero C, Sack M, Chandler J, Drossard J, Schuster F (1999). Transient expression of a tumor-specific single-chain fragment and a chimeric antibody in tobacco leaves.. Proc Natl Acad Sci USA.

[6] Sawers RJ, Farmer PR, Moffett P, Brutnell TP (2006). *In planta* transient expression as a system for genetic and biochemical analyses of chlorophyll biosynthesis.. Plant Methods.

[7] Li JF, Park E, von Arnim A, Nebenfuhr A (2009). The FAST technique: a simplified *Agrobacterium*-based transformation method for transient gene expression analysis in seedlings of *Arabidopsis* and other plant species.. Plant Methods.

[8] Wroblewski T, Tomczak A, Michelmore R (2005). Optimization of *Agrobacterium*-mediated transient assays of gene expression in lettuce, tomato and *Arabidopsis*.. Plant Biotechnol J.

[9] Zipfel C, Kunze G, Chinchilla D, Caniard A, Jones JD (2006). Perception of the bacterial PAMP EF-Tu by the receptor EFR restricts *Agrobacterium*-mediated transformation.. Cell.

[10] Conrath U, Pieterse CM, Mauch-Mani B (2002). Priming in plant-pathogen interactions.. Trends Plant Sci.

[11] Ryals JA, Neuenschwander UH, Willits MG, Molina A, Steiner HY (1996). Systemic acquired resistance.. Plant Cell.

[12] Vlot AC, Klessig DF, Park SW (2008). Systemic acquired resistance: the elusive signal(s).. Curr Opin Plant Biol.

[13] Felix G, Duran JD, Volko S, Boller T (1999). Plants have a sensitive perception system for the most conserved domain of bacterial flagellin.. Plant J.

[14] Schwessinger B, Zipfel C (2008). News from the frontline: recent insights into PAMP-triggered immunity in plants.. Curr Opin Plant Biol.

[15] Kunze G, Zipfel C, Robatzek S, Niehaus K, Boller T (2004). The N terminus of bacterial elongation factor Tu elicits innate immunity in *Arabidopsis* plants.. Plant Cell.

[16] Zipfel C, Robatzek S, Navarro L, Oakeley EJ, Jones JD (2004). Bacterial disease resistance in *Arabidopsis* through flagellin perception.. Nature.

[17] Pruss GJ, Nester EW, Vance V (2008). Infiltration with *Agrobacterium tumefaciens* induces host defense and development-dependent responses in the infiltrated zone.. Mol Plant Microbe Interact.

[18] Veena HJ, Doerge RW, Gelvin SB (2003). Transfer of T-DNA and Vir proteins to plant cells by *Agrobacterium tumefaciens* induces expression of host genes involved in mediating transformation and suppresses host defense gene expression.. Plant J.

[19] Erbs G, Silipo A, Aslam S, De Castro C, Liparoti V (2008). Peptidoglycan and muropeptides from pathogens *Agrobacterium* and *Xanthomonas* elicit plant innate immunity: structure and activity.. Chem Biol.

[20] Heese A, Hann DR, Gimenez-Ibanez S, Jones AM, He K (2007). The receptor-like kinase SERK3/BAK1 is a central regulator of innate immunity in plants.. Proc Natl Acad Sci USA.

[21] Spoel SH, Dong X (2008). Making sense of hormone crosstalk during plant immune responses.. Cell Host Microbe.

[22] De Vleesschauwer D, Djavaheri M, Bakker PAHM, Hofte M (2008). *Pseudomonas fluorescens* WCS374r-induced systemic resistance in rice against *Magnaporthe oryzae* is based on pseudobactin-mediated priming for a salicylic acid-repressible multifaceted defense response.. Plant Physiol.

[23] Bozsó Z, Ott PG, Szatmari A, Czelleng A, Varga G (2005). Early detection of bacterium-induced basal resistance in tobacco leaves with diaminobenzidine and dichlorofluorescein diacetate.. J Phytopathol.

[24] Conrath U, Beckers GJ, Flors V, Garcia-Agustin P, Jakab G (2006). Priming: getting ready for battle.. Mol Plant Microbe Interact.

[25] Rico A, Preston GM, Fatmi M (2008). *Agrobacterium* suppresses *P. syringae*-elicited salicylate production in *Nicotiana tabacum* leaves.. *Pseudomonas syringae* pathovars and related pathogens-Identification, epidemiology and genomics.

[26] Huang WE, Huang L, Preston GM, Naylor M, Carr JP (2006). Quantitative *in situ* assay of salicylic acid in tobacco leaves using a genetically modified biosensor strain of *Acinetobacter* sp. ADP1.. Plant J.

[27] Samuel MA, Hall H, Krzymowska M, Drzewiecka K, Hennig J (2005). SIPK signaling controls multiple components of harpin-induced cell death in tobacco.. Plant J.

[28] Koncz C, Schell J (1986). The promoter of T_L_-DNA gene *5* controls the tissue-specific expression fo chimaeric genes carried by a novel type of *Agrobacterium* binary vector.. Mol Gen Genet.

[29] Watson B, Currier TC, Gordon MP, Chilton MD, Nester EW (1975). Plasmid required for virulence of *Agrobacterium tumefaciens*.. J Bacteriol.

[30] Garfinkel DJ, Simpson RB, Ream LW, White FF, Gordon MP (1981). Genetic analysis of crown gall: fine structure map of the T-DNA by site-directed mutagenesis.. Cell.

[31] Stachel SE, Nester EW (1986). The genetic and transcriptional organization of the *vir* region of the A6 Ti plasmid of *Agrobacterium tumefaciens*.. EMBO J.

[32] de Torres-Zabala M, Truman W, Bennett MH, Lafforgue G, Mansfield JW (2007). *Pseudomonas syringae* pv. *tomato* hijacks the *Arabidopsis* abscisic acid signalling pathway to cause disease.. EMBO J.

[33] Melotto M, Underwood W, Koczan J, Nomura K, He SY (2006). Plant stomata function in innate immunity against bacterial invasion.. Cell.

[34] de Torres-Zabala M, Bennett MH, Truman WH, Grant MR (2009). Antagonism between salicylic and abscisic acid reflects early host–pathogen conflict and moulds plant defence responses.. Plant J.

[35] Spoel SH, Koornneef A, Claessens SMC, Korzelius JP, Van Pelt JA (2003). NPR1 modulates cross-talk between salicylate- and jasmonate-dependent defense pathways through a novel function in the cytosol.. Plant Cell.

[36] Koornneef A, Pieterse CMJ (2008). Cross talk in defense signaling.. Plant Physiol.

[37] Huang J, Cardoza YJ, Schmelz EA, Raina R, Engelberth J (2003). Differential volatile emissions and salicylic acid levels from tobacco plants in response to different strains of *Pseudomonas syringae*.. Planta.

[38] Hauck P, Thilmony R, He SY (2003). A *Pseudomonas syringae* type III effector suppresses cell wall-based extracellular defense in susceptible *Arabidopsis* plants.. Proc Natl Acad Sci USA.

[39] Chisholm ST, Coaker G, Day B, Staskawicz BJ (2006). Host-microbe interactions: shaping the evolution of the plant immune response.. Cell.

[40] Jones JD, Dangl JL (2006). The plant immune system.. Nature.

[41] Robinette D, Matthysse AG (1990). Inhibition by *Agrobacterium tumefaciens* and *Pseudomonas savastanoi* of development of the hypersensitive response elicited by *Pseudomonas syringae* pv. phaseolicola.. J Bacteriol.

[42] Flors V, Ton J, van Doorn R, Jakab G, Garcia-Agustin P (2008). Interplay between JA, SA and ABA signalling during basal and induced resistance against *Pseudomonas syringae* and *Alternaria brassicicola*.. Plant J.

[43] Nishimura MT, Stein M, Hou BH, Vogel JP, Edwards H (2003). Loss of a callose synthase results in salicylic acid-dependent disease resistance.. Science.

[44] Oh HS, Collmer A (2005). Basal resistance against bacteria in *Nicotiana benthamiana* leaves is accompanied by reduced vascular staining and suppressed by multiple *Pseudomonas syringae* type III secretion system effector proteins.. Plant J.

[45] Klement Z, Bozso Z, Kecskes ML, Besenyei E, Arnold C (2003). Local early induced resistance of plants as the first line of defence against bacteria.. Pest Manag Sci.

[46] Alvarez ME (2000). Salicylic acid in the machinery of hypersensitive cell death and disease resistance.. Plant Mol Biol.

[47] Mur LA, Kenton P, Atzorn R, Miersch O, Wasternack C (2006). The outcomes of concentration-specific interactions between salicylate and jasmonate signaling include synergy, antagonism, and oxidative stress leading to cell death.. Plant Physiol.

[48] Peng JL, Dong HS, Dong HP, Delaney TP, Bonasera JM (2003). Harpin-elicited hypersensitive cell death and pathogen resistance require the NDR1 and EDS1 genes.. Physiol Mol Plant Pathol.

[49] Ton J, Flors V, Mauch-Mani B (2009). The multifaceted role of ABA in disease resistance.. Trends Plant Sci.

[50] Lopez MA, Bannenberg G, Castresana C (2008). Controlling hormone signaling is a plant and pathogen challenge for growth and survival.. Curr Opin Plant Biol.

[51] Robert-Seilaniantz A, Navarro L, Bari R, Jones JD (2007). Pathological hormone imbalances.. Curr Opin Plant Biol.

[52] Clay NK, Adio AM, Denoux C, Jander G, Ausubel FM (2009). Glucosinolate metabolites required for an *Arabidopsis* innate immune response.. Science.

[53] Asselbergh B, Achuo AE, Höfte M, Van Gijsegem F (2008). Abscisic acid deficiency leads to rapid activation of tomato defence responses upon infection with *Erwinia chrysanthemi*.. Mol Plant Pathol.

[54] Laurie-Berry N, Joardar V, Street IH, Kunkel BN (2006). The *Arabidopsis thaliana JASMONATE INSENSITIVE 1* gene is required for suppression of salicylic acid-dependent defenses during infection by *Pseudomonas syringae*.. Mol Plant Microbe Interact.

[55] Uppalapati SR, Ishiga Y, Wangdi T, Kunkel BN, Anand A (2007). The phytotoxin coronatine contributes to pathogen fitness and is required for suppression of salicylic acid accumulation in tomato inoculated with *Pseudomonas syringae* pv. tomato DC3000.. Mol Plant-Microbe Interact.

[56] Zhao Y, Thilmony R, Bender CL, Schaller A, He SY (2003). Virulence systems of *Pseudomonas syringae* pv. *tomato* promote bacterial speck disease in tomato by targeting the jasmonate signaling pathway.. Plant J.

[57] Buell CR, Joardar V, Lindeberg M, Selengut J, Paulsen IT (2003). The complete genome sequence of the *Arabidopsis* and tomato pathogen *Pseudomonas syringae* pv. tomato DC3000.. Proc Natl Acad Sci USA.

[58] Huang WE, Wang H, Zheng H, Huang L, Singer AC (2005). Chromosomally located gene fusions constructed in *Acinetobacter sp*. ADP1 for the environmental detection of salicylate.. Environ Microbiol.

[59] Sambrook J, Russell DW (2001). Molecular cloning: A laboratory manual..

[60] Lee J, Klusener B, Tsiamis G, Stevens C, Neyt C (2001). HrpZ_Psph_ from the plant pathogen *Pseudomonas syringae* pv. phaseolicola binds to lipid bilayers and forms an ion-conducting pore *in vitro*.. Proc Natl Acad Sci USA.

[61] Rico A, Preston GM (2008). *Pseudomonas syringae* pv. tomato DC3000 uses constitutive and apoplast-induced nutrient assimilation pathways to catabolise nutrients that are abundant in the tomato apoplast.. Mol Plant-Microbe Int.

[62] Forcat S, Bennett MH, Mansfield JW, Grant MR (2008). A rapid and robust method for simultaneously measuring changes in the phytohormones ABA, JA and SA in plants following biotic and abiotic stress.. Plant Methods.

[63] Pfaffl MW (2001). A new mathematical model for relative quantification in real-time RT-PCR.. Nucleic Acids Res.

[64] de Torres M, Mansfield JW, Grabov N, Brown IR, Ammouneh H (2006). *Pseudomonas syringae* effector AvrPtoB suppresses basal defence in *Arabidopsis*.. Plant J.

