# Supplementary figures and images for: Agroinfiltration Reduces ABA Levels and Suppresses Pseudomonas syringae-Elicited Salicylic Acid Production in Nicotiana tabacum
Source: PLoS One. 2010 Jan 29;5(1):e8977. doi: 10.1371/journal.pone.0008977 (PMC2813289; doi:10.1371/journal.pone.0008977)

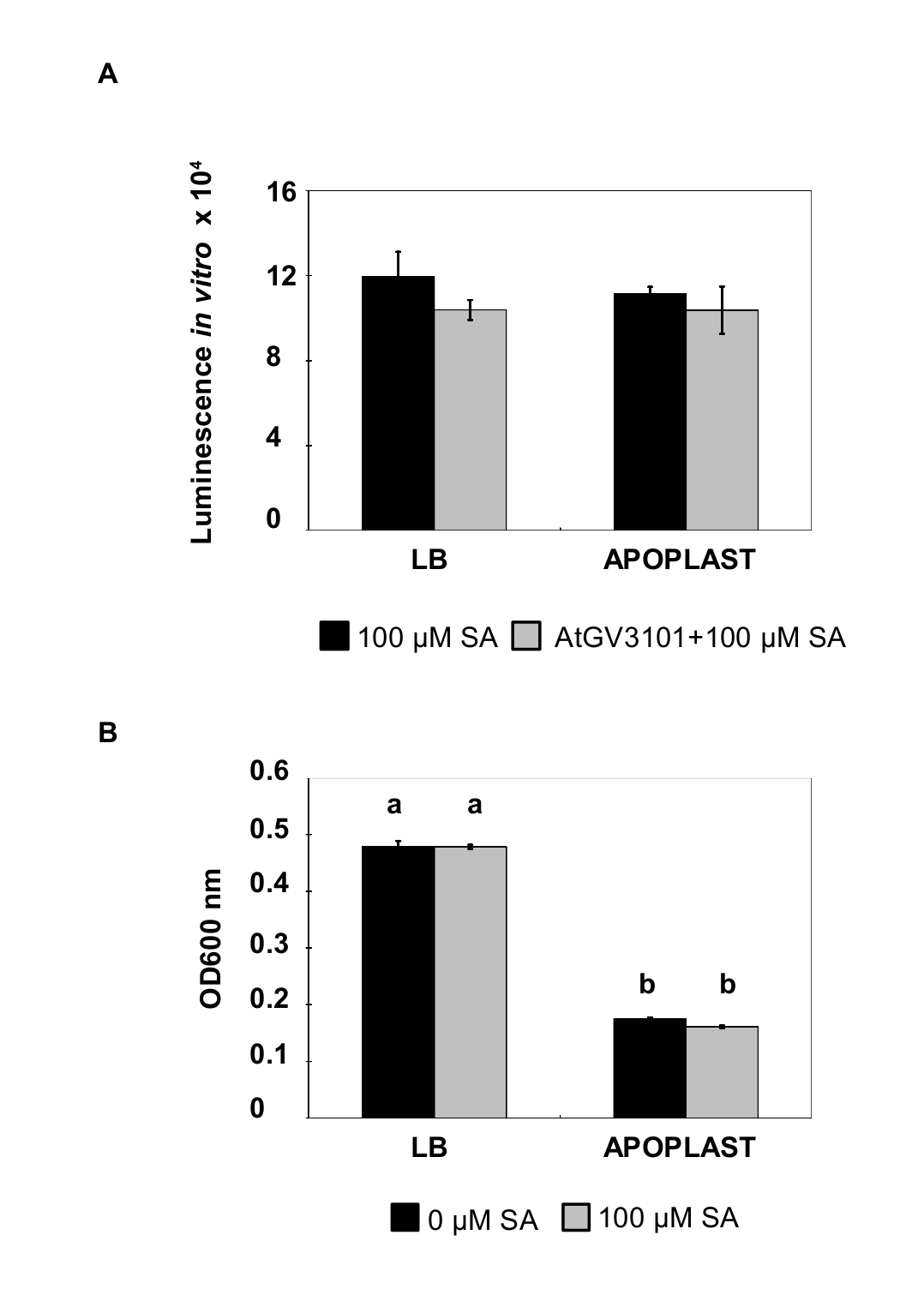

Supplement: Figure S1 — A. tumefaciens does not degrade SA. A. The ability of A. tumefaciens AtGV3101 (AtGV3101) to degrade SA was examined in plant extracts and synthetic media. AtGV3101 was inoculated into LB or tobacco apoplast extracts (APOPLAST) supplemented with 100 µM SA. After 24 h of growth, 20 µl of the supernatant of the AtGV3101 cultures was mixed with 50 µl of the SA biosensor ADPWH-lux (grey bars) and SA-induced luminescence was measured after 1 hour. As a control, the SA biosensor was mixed with uninoculated media containing 100 µM SA (black bars). General Linear Model (GLM) analysis did not reveal statistical differences between treatments (F = 0.7589; p = 0.5479; df = 11). The experiment was performed twice with similar results. B. The inability of A. tumefaciens AtGV3101 to degrade SA was not due to inhibition of bacterial growth by SA or plant extracts. A. tumefaciens AtGV3101 was inoculated into LB, tobacco apoplast extracts and the same media supplemented with 100 µM SA. Growth was assessed after 24 hours. General Linear Model (GLM) analysis revealed statistical differences between treatments (F = 1384.993; p<0.0001; df = 11). Means with the same letter were not significantly different at the 5% confidence level based on Tukey's Honestly Significant Mean Differences (HSD) Test. Error bars in A and B show standard error of the mean. (0.09 MB TIF) [file pone.0008977.s001.tif]

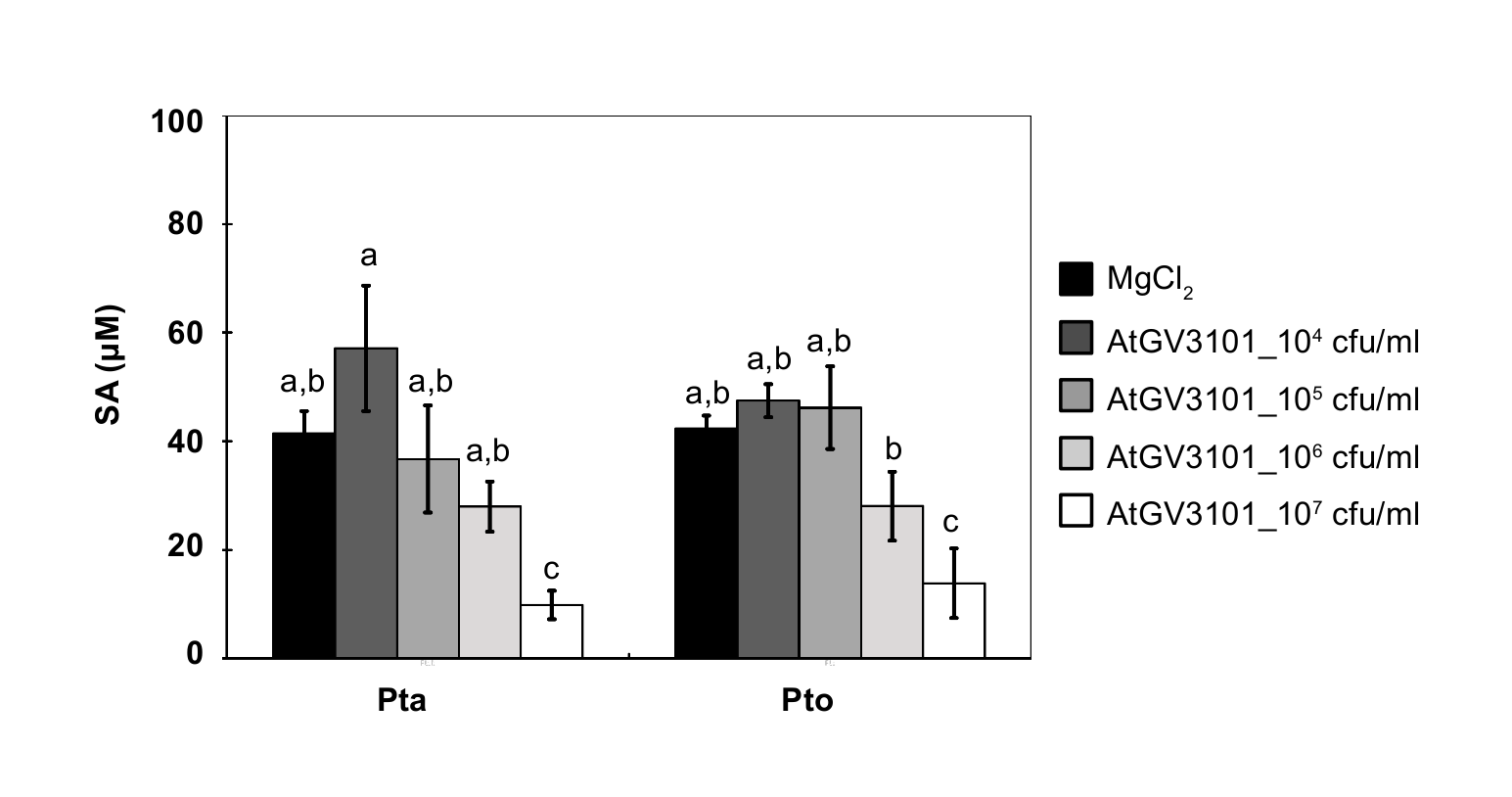

Supplement: Figure S2 — High densities of A. tumefaciens are needed to suppress P. syringae-elicited SA. Tobacco leaves were inoculated with A. tumefaciens GV3101 (AtGV3101) at 107, 106, 105 and 104 cfu/ml or 10 mM MgCl2(AS) (MgCl2) followed by inoculation with P. syringae pv. tabaci 11528 (Pta) or P. s. pv. tomato DC3000 (Pto) (105 cfu/ml) after 48 hours. The SA biosensor ADPWH-lux was inoculated into leaves 24 hours after infiltration with P. syringae. SA-induced luminescence was measured one hour after biosensor inoculation using a photon-counting camera and numerical SA values were calculated using a calibration curve as described in Huang et al. [26]. The chart shows average SA values from at least three leaves from different plants. Bars show standard error of the mean. (0.08 MB TIF) [file pone.0008977.s002.tif]

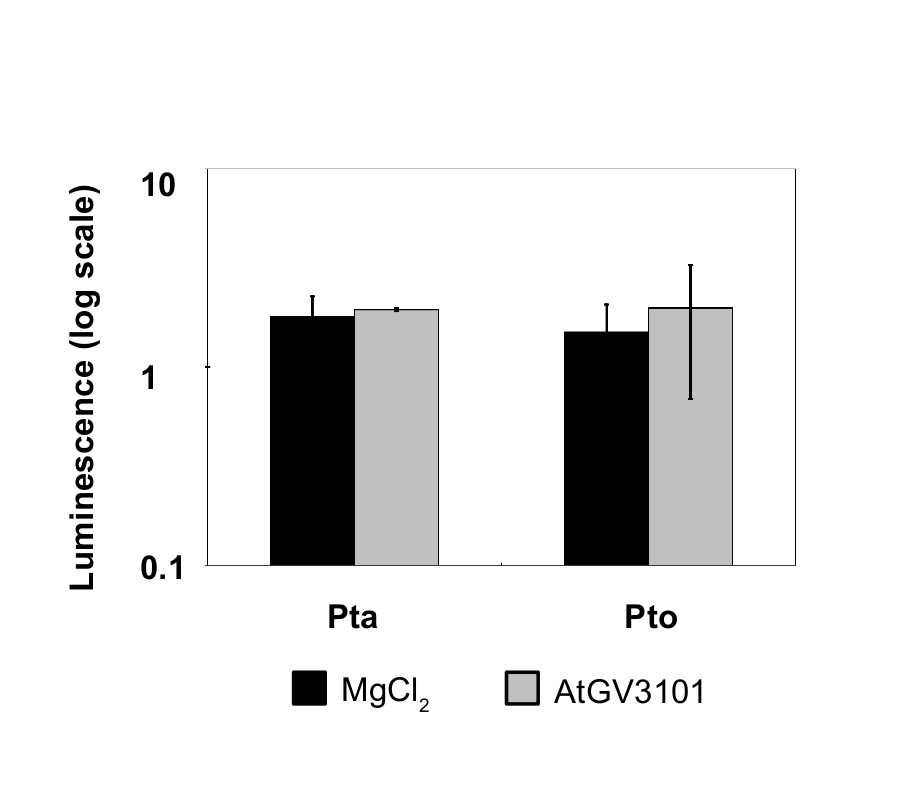

Supplement: Figure S3 — Co-inoculation of A. tumefaciens with P. syringae does not suppress P. syringae-elicited SA. Luminescence was examined 24 h after co-inoculation of 107 cfu/ml AtGV3101 with 105 cfu/ml Pta or Pto by infiltrating the ADPWH-lux SA biosensor in the inoculated zones. SA-induced luminescence was measured as described in Figure 2. The Y axis is shown in log scale. General Linear Model (GLM) analysis did not reveal statistical differences between treatments (F = 0.4547; p = 0.7283; df = 7). The experiment was performed twice with similar results. Error bars show standard deviation. (0.04 MB TIF) [file pone.0008977.s003.tif]
